# An improved map of conserved regulatory sites for *Saccharomyces cerevisiae*

**DOI:** 10.1186/1471-2105-7-113

**Published:** 2006-03-07

**Authors:** Kenzie D MacIsaac, Ting Wang, D Benjamin Gordon, David K Gifford, Gary D Stormo, Ernest Fraenkel

**Affiliations:** 1 Whitehead Institute for Biomedical Research, Nine Cambridge Center, Cambridge, MA 02142, USA; 2 MIT Computer Science and Artificial Intelligence Laboratory, 32 Vassar St., Cambridge, MA 02139, USA; 3 Department of Genetics, Washington University Medical School, St. Louis, MO 63110, USA; 4 Agilent Technologies, 245 1st Street, Cambridge, MA 02142, USA; 5 Biological Engineering Division, Massachusetts Institute of Technology, Cambridge, MA 02139, USA

## Abstract

**Background:**

The regulatory map of a genome consists of the binding sites for proteins that determine the transcription of nearby genes. An initial regulatory map for *S. cerevisiae *was recently published using six motif discovery programs to analyze genome-wide chromatin immunoprecipitation data for 203 transcription factors. The programs were used to identify sequence motifs that were likely to correspond to the DNA-binding specificity of the immunoprecipitated proteins. We report improved versions of two conservation-based motif discovery algorithms, PhyloCon and Converge. Using these programs, we create a refined regulatory map for *S. cerevisiae *by reanalyzing the same chromatin immunoprecipitation data.

**Results:**

Applying the same conservative criteria that were applied in the original study, we find that PhyloCon and Converge each separately discover more known specificities than the combination of all six programs in the previous study. Combining the results of PhyloCon and Converge, we discover significant sequence motifs for 36 transcription factors that were previously missed. The new set of motifs identifies 636 more regulatory interactions than the previous one. The new network contains 28% more regulatory interactions among transcription factors, evidence of greater cross-talk between regulators.

**Conclusion:**

Combining two complementary computational strategies for conservation-based motif discovery improves the ability to identify the specificity of transcriptional regulators from genome-wide chromatin immunoprecipitation data. The increased sensitivity of these methods significantly expands the map of yeast regulatory sites without the need to alter any of the thresholds for statistical significance. The new map of regulatory sites reveals a more elaborate and complex view of the yeast genetic regulatory network than was observed previously.

## Background

Transcription factors are proteins that regulate an organism's genetic program by binding to specific sites in the genome and modifying the expression of nearby genes. Mapping these sites is an important step in understanding transcriptional regulation, and can be significantly facilitated by integrating multiple data sources such as sequence, gene annotations, and phylogenetic conservation [1,2]. A previously published study [3] reported an initial regulatory map for *Saccharomyces cerevisiae *by analyzing genome-wide chromatin immunoprecipitation (ChIP) data for 203 proteins. Harbison and co-workers used motif discovery programs in an effort to detect statistically over-represented sequence patterns (motifs) in the bound regions that were likely to correspond to the binding specificity of the immunoprecipitated proteins. Applying six different algorithms, they identified thousands of motifs. After extensive filtering and statistical testing, they reported high-confidence results for sixty-five proteins. They used these high-confidence motifs to identify sites that were in regions bound by the corresponding protein and that were conserved across at least 3 yeast species. We wished to expand and refine the yeast regulatory map by using a more sophisticated incorporation of phylogenetic conservation information.

Recently, many authors have reported algorithms for motif discovery that use evolutionary conservation. Kellis *et al*. presented a computational method involving the genome-wide discovery of a catalogue of conserved motifs, which they annotated by searching for overrepresented functional categories among the genes with each motif [4]. Several programs use an expectation maximization-based search over a probability model of DNA sequence to find conserved motifs. EMnEM [5] and PhyME [6] both incorporate probabilistic evolutionary models into EM-based motif searches. Several other approaches to using conservation information in motif discovery use Gibbs sampling to sample a probability space and search for motifs. CompareProspector is a Gibbs sampling algorithm that uses a pre-computed score to measure the conservation level across windows in sequence alignments, and then biases the motif search to regions that are highly conserved [7]. PhyloGibbs is another conservation-based Gibbs sampling algorithm that leverages conservation by assuming the motif must be present in all species in a conserved region [8]. Recently, another Gibbs sampler was developed to incorporate phylogenetic data by employing two substitution matrices for motif instances and background sites, with the background model estimated from orthologous sequence alignments and the motif model assuming half the branch length of the background model [9]. All these algorithms have been demonstrated, in certain contexts, to outperform similar methods that don't take advantage of conservation information.

Tompa and co-workers [10], who recently assessed a number of motif discovery programs, demonstrated that these algorithms perform much better on synthetic data than on real data. Their results highlight the importance of evaluating algorithms using experimental datasets such as those of Harbison *et al*. Using motif discovery programs to identify the specificity of proteins from experimental data is particularly challenging because there are many statistically significant motifs in such data, and no guarantee that a motif that corresponds to a factor's specificity will be highly ranked, or even discovered. Harbison *et al*., who used six separate motif discovery programs, observed that each motif discovery program found the correct motif for at least one protein that was not found by the other methods. However, no single program demonstrated a clear superiority (D. Benjamin Gordon, personal communication). Their analysis provides a useful benchmark for evaluating motif discovery approaches on experimental data.

In this study, we report two improved algorithms for conservation-based motif discovery, Converge and PhyloCon, and we use these methods to reanalyze the data of Harbison *et al*. Using statistical tests identical to the ones used by Harbison *et al*, we find that Converge and PhyloCon each identify more correct motifs than were found using the combined results of the six programs employed in the earlier study. The motifs discovered by Converge and PhyloCon are often complementary. Combining these motifs, we were able to significantly expand the map of yeast regulatory sites without the need to alter any of the thresholds for statistical significance. The new map reveals a more elaborate and complex view of the yeast genetic regulatory network than was observed previously. The updated map can be viewed and downloaded from the authors' website [11].

## Results

### Algorithmic overview and improvements

PhyloCon and Converge are both designed to find evolutionarily conserved motifs among a set of genes that are believed to be co-regulated. These two programs use different inputs, search algorithms and scoring statistics. PhyloCon [13] begins with unaligned sequences and generates many local alignments from each orthologous group. The local alignments are assembled using a greedy algorithm to identify patterns that are both conserved in orthologous genes and present in many of the co-regulated promoters. By contrast, Converge [3] searches over pre-computed, static alignments. Converge is based on an expectation-maximization (EM) algorithm [14,15] that has been modified to include conservation in the joint probability model. Converge motifs are scored by comparing the frequency of matching sequences in the bound and not-bound genes using a hypergeometric distribution. The previously published version of PhyloCon scores sequences using the ALLR statistic [13], which measures the relative likelihood that a sequence would emerge from the motif model and the background sequence model (see Methods).

We made several modifications to the previously published PhyloCon and Converge algorithms for this study (see Methods). The most important modification for Converge was the introduction of a phylogenetic model that allows for different evolutionary distances between each species and the primary genome (*S. cerevisiae*). We modified the core EM algorithm to dynamically adjust these distances during motif discovery. Thus, Converge simultaneously discovers motifs and their evolutionary history, and it is able to detect cases where the sets of genes bound by a particular regulator have diverged in the species being studied. To improve the performance of PhyloCon, we introduced a new scoring statistic, the Total Log Likelihood Ratio (TOLLR). This score limits the overfitting of PhyloCon motifs to datasets that are expected to contain a significant number of false positives.

### Motif discovery performance

**Figure 1:**
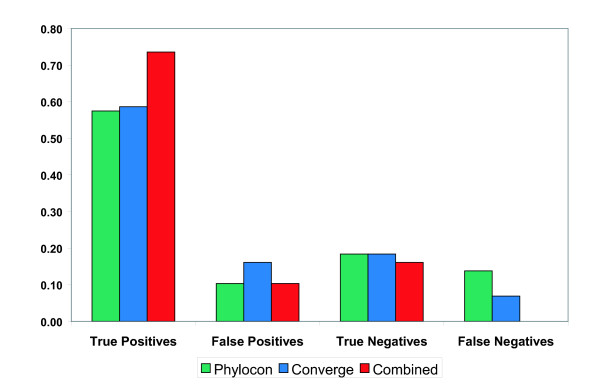
**Performance of PhyloCon, Converge, and the combined motif set on data for factors of known specificity**. PhyloCon and Converge both recover more true positives than the suite of 6 programs employed in Harbison *et al*. Combining the results of PhyloCon and Converge significantly increases the number of true positives recovered, and eliminates false negatives, without a large adverse effect on the false positive rate. For definitions of the scoring criteria, see the Methods section.

PhyloCon and Converge each showed significantly better performance than the combined results from the six programs used in Harbison *et al*. [3]. Using the same approach as that study, we scored the motifs produced by each program using empirical p-values. The top-ranked motif from each program was accepted as the predicted specificity for the corresponding protein if it had a p-value < 0.001. We assessed the performance of PhyloCon and Converge using a set of 87 yeast transcription factors for which the specificity has been reported in the literature (see Additional file 1). In Harbison *et al*., the predicted specificities derived from a combination of six programs matched the known specificities for 44 of the 87 proteins (51%). By contrast, PhyloCon produced 50 true positives (57%) and 9 false positives (10%), and Converge found 51 true positives (59%) and 14 false positives (16%). PhyloCon and Converge were unable to find statistically significant motifs for 28 (32%) and 22 (25%) of these factors respectively (Figure 1).

Combining results from PhyloCon and Converge allowed us to expand the set of discovered motifs without significantly degrading performance for the factors with known specificities. The number of true positives increases to 64 (74%), with 9 false positives (10%), 14 true negatives (16%), and 0 false negatives. (The criteria for merging PhyloCon and Converge results as well as the criteria for classifying motifs into these four categories are described in the Methods section).

### Updated catalog of yeast factor specificities

**Figure 2:**
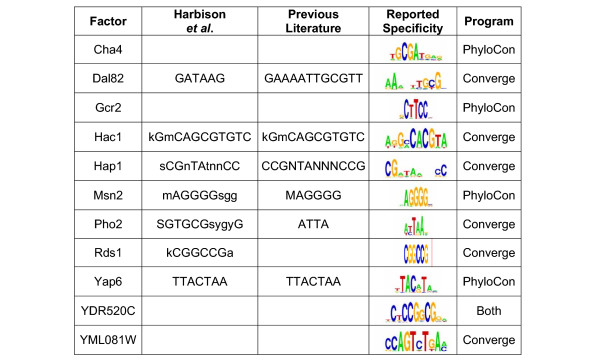
Selected Factor Specificities in the New Yeast Regulatory Map.

Combining the results from two conservation-based motif discovery programs allowed us to significantly increase the number of transcription factors for which we could predict binding specificities with high-confidence. Of the 172 factors investigated (all those that bound at least 4 probes), we discovered statistically significant motifs (p < 0.001) for 98 factors, 64 of which had experimentally determined specificities reported in the literature. The combined results of PhyloCon and Converge discover 33 more motifs than were found by Harbison and co-workers, who used the same strict selection criteria. Of the 98 motifs, 43 were discovered by both programs, 22 were found only by PhyloCon, and 33 were discovered only by Converge. In some of the cases where no motif was found but the protein had a known specificity, the input sequences contained few regions that matched that specificity. In other cases, very few probes had been bound by the protein. The discovered motifs were augmented with 26 factor specificities from the literature, to produce a final catalogue of 124 yeast transcription factor binding specificities. The complete list of discovered motifs is provided in Additional file 2, and several examples are shown in Figure 2. In some cases, the new motifs differ substantially from the motifs reported in Harbison *et al*. For example the specificity discovered previously for Pho2 was SGTGCGsygyG. Our analysis predicts a specificity of AYTAAr. The new motif is more consistent with the results of gel shift and DNAse footprint analysis [16] and with the fact that that Pho2 encodes a homeodomain protein [17], a class of transcription factors that tend to bind to AT-rich sequences. The factor Dal82 is now predicted to have a specificity of AAaNwTgyG, consistent with previously reported experimental evidence [18]. The motif reported in Harbison *et al*. (GATAAG) is likely to represent the binding specificities of Gln3, Gat1, and Dal80, which are known to co-regulate allophanate/oxalurate-dependent genes along with Dal82 [19]. No motif was previously reported for the zinc cluster protein YDR520C. The motif that we discover contains a palindromic CGG repeat, consistent with the expected specificity for a zinc cluster protein [20-22].

### An updated regulatory map for yeast

**Figure 3:**
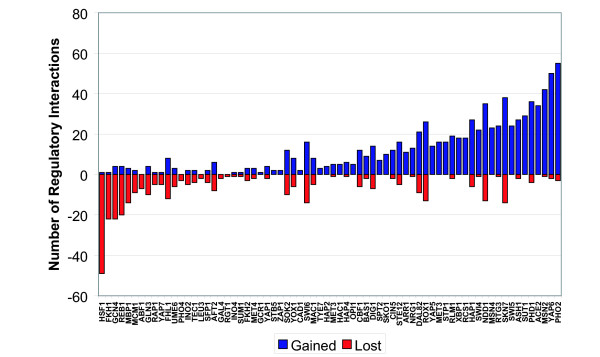
**Changes in the number of putative regulatory interactions for factors common to the old and new regulatory codes**. For each modified motif, the number of regulatory interactions added and lost relative to the previously reported map is shown. Our analysis produced modified factor binding specificities for 85 factors, resulting in a net gain of 398 putatively regulated genes.

**Figure 4:**
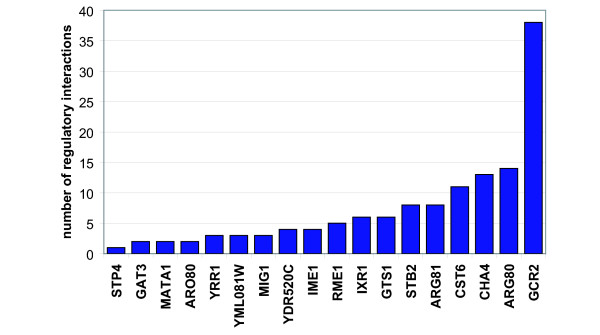
**Regulatory interactions added through the addition of new factor specificity estimates**. A total of 200 genes were identified as being putatively regulated by factors with newly reported motifs.

Using the new catalogue of yeast specificities, we are able to build a more complete and comprehensive regulatory map for *Saccharomyces cerevisiae*. We scanned the *S. cerevisiae *genome for putative regulatory interactions using our updated motif catalogue and the same criteria used by Harbison *et al*. As in that study, we restrict our analysis to the highest confidence sites, defined as those containing conserved motif matches that were bound by the corresponding factor at a p-value < 0.001. The new map contains a total of 4229 conserved and bound motif sites across 2022 genes, compared to the 3353 sites across 1883 genes in Harbison *et al*. The new and the old sets of motifs have similar information content (mean information content of 11.77 bits and information content per base of 1.24 bits in the new code, compared to 11.10 bits and 1.25 bits in the old code), suggesting that this increase is not due to an overall loosening of the specificity estimates. Figure 3 and Figure 4 show the change in the number of bound genes by factor between the new and old maps. The net gain in the number of putative regulatory interactions between transcription factors and proteins is 636, with 133 of these accounted for by new binding specificity estimates for 18 factors that had no previously reported motif.

The improved motifs reveal regulatory interactions for a number of transcription factors that are consistent with their known functions. For example, the refined motif for Msn2 detects regulatory sites in 39 genes that were not detected in the previous study. Msn2 is known to function in the transcriptional response to stress [23]. Of the newly identified targets, there is a significant (p < 0.01) over-representation of genes with the GO annotation "stress-response". Similarly, the refined Xbp1 motif results in a gain of 18 regulatory interactions. The new targets are enriched at a p-value < 0.02 for genes with the GO annotation "morphogenesis", consistent with a previously reported regulatory role for this transcription factor [24].

The revised map also provides new insights into the regulatory roles of several transcription factors. For example, the revised motif for Hap1 reveals that this transcription factor has an extensive role in regulating synthesis of ergosterol, a fungal-specific pathway that is a target for anti-fungal drugs. The previous map revealed regulatory interactions of Hap1 with genes for the ergosterol biosynthetic enzymes Erg5, Erg9 and Erg11. In the new map, we find interactions with genes for six additional enzymes in this pathway: Erg2, Erg8, Erg10, Erg25, Faa1, and Hmg1. In addition, the new map details an expanded role for Hap1 in regulating expression of components of the electron transport chain. Regulatory interactions with genes for two components of the cytochrome c oxidase complex, Cox7 and Cox8, were added to the three already present (Cox4, Cox6, and Cox13). A regulatory interaction with the gene for Qcr6, a component of ubiquinol cytochrome c reductase, was added to the previously reported interaction with the gene for Cor2, also a member of this complex. Finally, a Hap1 regulatory interaction with cytochrome c isoform 2, Cyc7, was added to previously discovered interactions with genes for three other cytochromes, Cyc1, Cyb2, and Cyt1, in the old regulatory code.

### Network analysis

**Figure 5:**
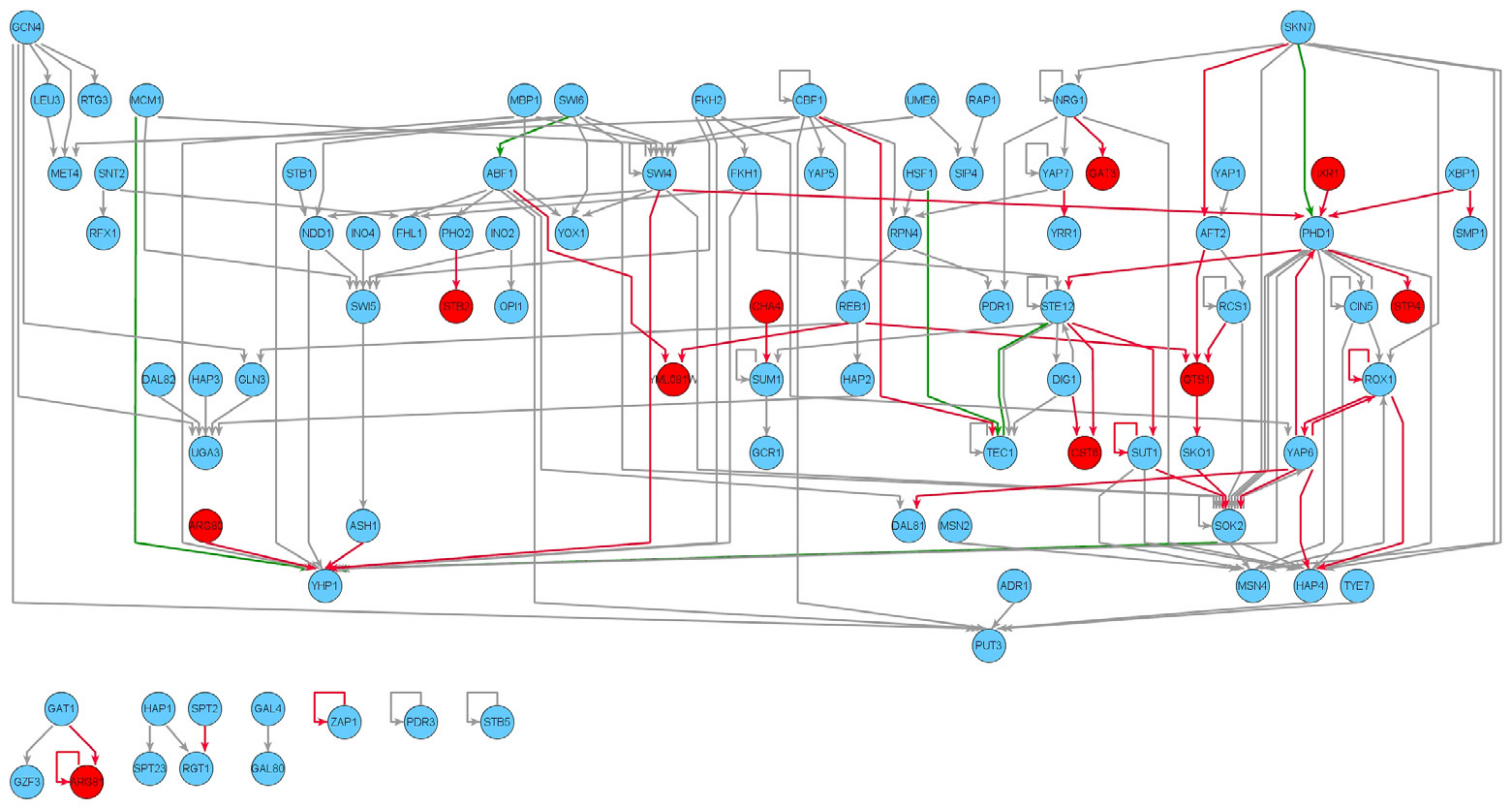
**Yeast transcriptional regulatory network**. Nodes correspond to transcription factors and an edge from one factor to another indicates that the first factor regulates the second. Red nodes correspond to factors without a previously reported specificity. Edges are colored red for interactions unique to the new map, grey for interactions common to the old and new maps, and green for interactions unique to the old map. There are 39 new interactions gained and 6 interactions lost relative to the previous map.

We examined the network of regulatory interactions between transcription factors in order to understand the system-level implications of our improved map. The previously reported regulatory code and the revised code were used to generate interaction networks for all the yeast transcription factors (Figure 5). The new map results in a striking increase in the complexity of the yeast regulatory network. Thirty-nine new interactions are reported in the network, with six interactions lost from the previous version. We searched this network for occurrences of six regulatory network motifs: autoregulation, feed-forward regulation, multi-component loops, single-input, multi-input, and regulatory chains, as described in [25]. Table 1 lists the number of each regulatory motif in the new and old networks. There is an increase in the number of all six regulatory motif types, with a particularly striking increase in the number of regulatory chain motifs, owing to the motif's combinatorial dependence on the total number of interactions in the network. The overall picture that emerges from this analysis is of a more complex interplay of transcription factor influences in yeast regulatory networks than could be deduced from the previously reported regulatory code.

The network based on the new map reveals several cases of feedback regulation that were not present in the previous version. The regulators Arg81, Rox1, Sut1, and Zap1 are all found to have an autoregulatory interaction in the new map. Of these, Rox1 [26] and Zap1 [12] have been previously shown to regulate their own expression. The map also contains evidence of enhanced roles for a number of factors in the yeast transcriptional regulatory network. With its updated specificity, Yap6 now appears to act as a regulatory hub, displaying five new interactions with transcription factors, three of which (Phd1, Sok2, and Hms2) are involved in pseudohyphal differentiation [27-29]. The stress-induced factor Xbp1, previously implicated in cell-cycle function [30], now displays interactions with the pseudohyphal determinant Phd1, and Smp1, a factor required for cell viability in the stationary phase [31].

### Complementarity of motif discovery programs

PhyloCon and Converge each find motifs that are missed by the other program. This complementarity arises from differences between the programs in (1) optimization criteria and (2) underlying evolutionary assumptions.

#### Optimization criteria

The programs search for motifs that maximize different metrics: the enrichment and ALLR scores (see Methods). As a result, motifs judged significant by one program can be ranked poorly by the other. In 11 of the 15 cases where the correct motif was discovered solely by PhyloCon, Converge found the same motif, but with a poor enrichment score. Similarly, of the 15 correct motifs reported only by Converge, seven were also discovered by PhyloCon but only with ALLR scores at significance level P < 0.01, and five more were discovered at significance level P < 0.02.

#### Evolutionary assumptions

PhyloCon dynamically realigns orthologous sequences, making no assumptions regarding the relative location of binding sites. However, it assumes that the sequences from each species should contribute equally to motif discovery. Converge, by contrast, assumes that the position of binding sites will be aligned in the orthologous sequences, but it makes no assumptions about the importance of the sequences from each species.

The consequences of these differing assumptions can be seen by examining the results for Xbp1 and Rds1. The Xbp1 motif is present in 53% of the *S. bayanus *sequences orthologous to the bound *S. cerevisiae *probes, but only 27% of the *S. cerevisiae *motif matches align with a match in *S. bayanus*. By dynamically realigning the orthologous sequences, PhyloCon discovers the Xbp1 motif, while Converge is unable to learn the correct specificity. In the case of Rds1, the situation is reversed: Converge finds the motif, while PhyloCon does not. In this case, Converge determines that there is a very low probability that a match to the Rds1 motif will occur in *S. bayanus *in positions that contain the motif in *S. cerevisiae*. The Converge parameter θ_k_, which measures the genome-wide probability of observing a motif in aligned genome *k *when it is present in the primary genome, falls to 0.058 (see Methods). As a result, the *S. bayanus *sequences have almost no influence on the discovered motif. It is worth noting that the Rds1 protein from *S. bayanus *is only 32% identical to its *S. cerevisiae *ortholog, compared to approximately 72% for other transcription factors in these two species. These data suggest that in *S. bayanus *Rds1 does not regulate the orthologs of the genes that are bound by Rds1 in *S. cerevisiae*, and that both the protein and its former binding sites have evolved.

## Discussion

In this study we have demonstrated, on a large scale and with real data, how the use of phylogenetic conservation information can improve the ability to learn transcription factor binding specificities and paint a more detailed picture of gene regulation in yeast. In Harbison *et al*., the authors presented a first draft of the regulatory code for a eukaryotic organism and speculated that future revisions might arise out of the collection and analysis of new experimental data, or through the use of new computational algorithms that integrate different data sources. In this work we have presented a revised regulatory code by combining the results of two complementary algorithms that integrate sequence and conservation data to discover sequence motifs. The resulting map provides a broader picture of regulatory programs in yeast.

Using Motif Discovery algorithms to discover the specificity of transcription factors from experimental data is a challenging problem. The data of Harbison *et al*. are particularly useful for evaluating how motif discovery algorithms perform for this purpose. Aside from the original study and our current results, we are aware of only one other paper that has attempted to identify binding specificities from these data. Li and Wong reported a conservation-based motif discovery program, which they refer to as a tree sampler, that they applied to many of the same datasets that are all included in our analysis [32]. We compared our results to the published results of Li and Wong, which we downloaded directly from their publication (see Additional file 3). Applying the same criteria to the results of all three programs, we find that tree sampler identified correct motifs in 39 of the 53 (74%) cases reported, while PhyloCon identified 44 of these motifs (83%), and Converge correctly identified 43 (81%). We note that in their paper Li and Wong report worse results for PhyloCon than we obtained. The differences may be due to the fact that they used an earlier version of PhyloCon or ran it with non-optimal parameters.

A sound and principled use of conservation information allowed both PhyloCon and Converge to perform well on these data. Both programs outperformed the tree sampler and they each recovered more known factor binding specificities than a suite of six other programs combined. PhyloCon and Converge use complementary approaches to incorporate phylogenetic conservation information into motif discovery. Converge reduces its search space by assuming that alignments are high quality and static, whereas PhyloCon makes no such assumption and dynamically aligns orthologous sequences. Converge weights each genome differently and learns these weightings during motif discovery, whereas PhyloCon weights all orthologous sequences equally. Finally, PhyloCon searches for motifs by optimizing the ALLR score, whereas Converge selects EM starting points, and evaluates the resulting motifs using a hypergeometric enrichment score. Because of these differences, each program finds some motifs that are missed by the other one. Combining the results of these programs leads to a significant elaboration of the yeast regulatory code.

There is increasing interest in using motif discovery algorithms to discover the binding specificity of proteins from high-throughput data. However, it is important to recognize the limitations of these methods, which rely largely on statistical criteria. For example, some proteins are known to bind DNA indirectly through interactions with other proteins. Gal80 is an inhibitor of Gal4 that binds the Gal4 protein and lacks a DNA-binding domain. The ChIP-chip data reveal that they have many common targets. Our motif discovery algorithms identify the known Gal4 motif as the specificity of both proteins. Given the known physical interactions of Gal4 and Gal80, these data imply that Gal80 is directed to its targets indirectly through its association with Gal4. Similarly, Gcr2, which is known to act together with Gcr1 to regulate glycolysis genes [33], has a newly reported specificity that matches the known specificity of Gcr1, suggesting that Gcr2 may be recruited to the DNA through interactions with Gcr1. Additional sources of biological data will need to be incorporated into algorithms to determine whether a motif represents the specificity of a protein or an interacting factor.

## Conclusion

We have demonstrated a practical approach to analyzing experimental data by combining two complementary motif discovery programs that use phylogenetic conservation. We anticipate that progress in mapping the architecture of regulatory programs in eukaryotes will arise from a more thorough understanding of the relative merits of various approaches to motif discovery, as well as algorithmic developments in integrating various data sources. Algorithms that make use of phylogenetic conservation, factor homology, positional information, and other prior information sources will become more and more important as we attempt to apply motif discovery methods to higher eukaryotes. However, since no algorithmic approach to motif discovery has demonstrated a clear superiority across all applications, it will become equally important to integrate various motif discovery methods in a more intelligent manner [34]. Ultimately, unraveling the regulatory code of higher eukaryotes may be greatly facilitated by a 'mixture of experts' approach to motif discovery that uses the output of multiple algorithms, each intelligently integrating various data sources in unique ways, to generate consensus binding motifs for a factor of interest.

## Methods

### Motif discovery

The Converge and PhyloCon programs were applied separately to the chromatin immunoprecipitation data described in [3]. There are a total of 308 experiments for 172 factors in which at least four probes are bound with p-value cutoff of 0.001. Alignments of these probe sequences with three additional yeast species, *S. paradoxus*, *S. mikatae*, and *S. bayanus *were provided as input to Converge and the orthologous sequences from all four species were provided to PhyloCon.

### Motif discovery with converge

Converge uses phylogenetic conservation information from high quality sequence alignments to improve the performance of motif discovery. The input to the algorithm consists of a series of sequences believed to share a common motif, which we will refer to as probe sequences, and any available pair-wise alignments of these probes to orthologous sequences from related species. In the underlying model for Converge, the probability that a motif occurs at a particular position in a probe depends not only on the sequence of the probe, but also on the sequence of the corresponding positions in all of the available aligned orthologs, as explained below. Motifs are discovered using the Expectation-Maximization algorithm. We based our implementation of EM in large part on the ZOOPS model of Bailey and Elkan [14,15], but used a probability model that incorporates data from the orthologous sequences. An early version of this program was used in [3]. A complete description of the algorithm is provided in the Additional file 4.

#### Selection of seeds for the converge algorithm

Since the EM algorithm performs a local optimization, the motifs that are discovered depend on the initialization conditions. We generated initialization seeds for all data sets at motif widths of 6, 8, 10, 15, and 20 base pairs. For motif widths less than or equal to 10, we selected seeds by first identifying the top 400 n-mers in the data set. We calculated a rough conservation score for each n-mer by counting the total number of bases where the sequence was conserved across all intergenic regions in at least 50% of the aligned *sensu stricto *yeast species. We associated a p-value with these scores by fitting the result to a binomial distribution, or when the number of occurrences was sufficiently large, to a normal approximation to the binomial distribution. We discarded all n-mers with a conservation p-value greater than 0.1 from consideration as seeds. The remaining n-mers were scored using the hypergeometric distribution to give an enrichment p-value associated with observing the same, or greater, number of n-mer occurrences in a randomly selected, equally sized, sample of probe sequences in *S. cerevisiae*. We selected the 20 most statistically enriched conserved n-mers as seeds.

For motif widths greater than 10, we used a gapped model where an n-mer consisted of two flanking regions of specified sequence, with the central region allowed to take on any sequence. This approach was intended to compensate for the paucity of very large n-mers with multiple occurrences in the data sets. Also, many transcription factors are known to bind specific sequences separated by non-specific regions of DNA and it was hoped that this seeding approach would help in the discovery of such motifs. Each flanking region was set to a size equal to one third of the motif width, rounded down. The top 400 gapped n-mers were identified and subjected to the same conservation criterion described above. We scored these gapped n-mers for enrichment and the top 20 were selected as seeds, with the gapped region initialized to background base frequencies for use in EM.

#### Expectation maximization

Converge treats a given pair-wise sequence alignment as arising from a mixture of probability models. The primary sequence is modelled as a mixture of a 4th order Markov background and a position-specific scoring matrix (PSSM) representation of the motif region. A given sequence is assumed to contain either one or zero motifs. The sequences aligned to the primary sequence are constrained such that they may contain a motif only when a motif is also present in the primary genome. Aligned genomes are weighted using a parameter θ, which is the probability of observing a motif in the aligned genome when a motif is present in the primary genome. This parameter, which is shared across all instances of a motif in a particular genome, is updated iteratively over the course of the algorithm.

Regions in the sequence alignments that contain gaps in the primary genome are expunged, since only motifs in the primary genome are of interest. Sequence regions in the motif window of supporting genomes are modelled as a mixture of two PSSMs: one that incorporates gaps and one that doesn't. This allows regions without gaps in the aligned sequences to be weighted differently than gapped regions during the motif search and allows Converge to take advantage of the information present in gaps in the alignments. The joint probability model describing the sequence alignments is:

log *P*(**X**,**G**,**Z **| **Ψ**) = log *P*(**X **| **Z**,**G**,**Ψ**) + log *P*(**G **| **Ψ**) + log *P*(**Z**_1...*k *_| **Z**_0_,**Ψ**) + log *P*(**Z**_0 _| **Ψ**)

The data is modelled as a joint density over observed data (**X **and **G**) and hidden data (**Z**). **X **represents the sequences, **G **is a vector of binary variables that indicate whether a gap is observed in the motif window in the aligned genomes, and **Z **is a second vector of binary variables that indicate motif locations. The motif PSSM, genome weightings, and gapped region weightings are subsumed in the parameter vector **Ψ**. A motif is assumed to only occur in the aligned sequences when it is present at the same position in the primary genome. Therefore the motif position indicator variables for the aligned sequences, Z_1...k_, are dependent on the value of the indicator variable in the primary genome, Z_0_. All gaps in the primary genome are removed in a pre-processing step.

#### Converge motif discovery implementation details

For each seed sequence in a data set, we ran the Converge algorithm until the mean squared difference between motifs in subsequent iterations was less than 10^-3 ^for each position in the PSSM, and the value of each θ parameter changed by less than 10^-3^. In the M-step, we add 0.01 pseudo counts at each position in the PSSM. We used an estimate of the prior probability of motif occurrence in a given probe of 0.2 and set its learning rate to 0.5. The θ parameter was initialized to a simple measure of phylogenetic distance between the aligned species and *Saccharomyces cerevisiae*: the mean number of matches per position relative to *S. cerevisiae *in all probe alignments. This gave θ initialization values of 1.00, 0.80, 0.63, and 0.58 for *S. cerevisiae*, *S. paradoxus*, *S. mikatae *and *S. bayanus*, respectively. We estimated background sequence probabilities using a 4th order Markov model calculated separately for each species from its set of intergenic regions. The implementation of Converge was written in Python, with the computationally intensive EM subroutines written in C++.

### Motif discovery with PhyloCon

"PhyloCon" stands for Phylogenetic Consensus. This algorithm is specifically designed for regulatory motif discovery when both phylogenetic information and gene co-regulation information are available. Here we briefly describe the original algorithm [13] and several algorithmic improvements we made to accommodate this study.

The input provided to PhyloCon is a collection of promoter sequences from a species, together with orthologous sequences. For each group of orthologous sequences, PhyloCon first tries to generate many local, ungapped multiple sequence alignments by applying the wconsensus algorithm [35]. These alignments, including the optimal one and many sub-optimal ones, are converted to profiles that represent conserved regions in the promoters. PhyloCon then compares profiles generated from different orthologous groups and identifies ungapped high scoring local alignments between any two profiles. The alignment uses a Smith/Waterman-style dynamic programming algorithm, and the scoring function for aligning two positions from two profiles is the "average log likelihood ratio" (ALLR) statistic, described below. Two promoters bound by the same transcription factor, often have their binding sites optimally aligned in high scoring pairs (HSPs). Once a HSP is determined, the parental profiles are merged, and a new profile is created according to the HSP. Such newly generated profiles represent the shared portion between two orthologous groups, presumably containing the shared motif. PhyloCon then compares these profiles to other new profiles that contain non-overlapping orthologous groups, as well as to profiles that represent the initial conserved alignments of single orthologous group. HSPs between any two comparable profiles are found and new profiles are generated. PhyloCon continues profile comparison cycles until no new profile can be created from existing profiles. By using the ALLR statistic, PhyloCon is often able to precisely locate the boundaries of common sections between two profiles. Therefore, unlike most other motif finders, PhyloCon does not require the length of the motif *a priori*.

#### The ALLR statistic

The ALLR is a recently developed, powerful statistic for hypothesis testing [13]. Given two profiles, it compares the joint probability of observing one profile's training data given the log likelihood ratio of the other profile with the background distribution. This statistic is, therefore, a useful one to determine whether profiles derived from distinct sets of sequences should be merged.

For profile comparison, the ALLR is implemented in the following manner. A profile is essentially a string of columns, each column being a distribution vector f_b _= {f_A_, f_C_, f_G_, f_T_}, which represents the estimation of base frequencies at this position; n_b _= {n_A_, n_C_, n_G_, n_T_} denotes the observed base count at this position; p_b _= {p_A_, p_C_, p_G_, p_T_} denotes background base frequencies. A pseudo-count proportional to prior base frequency is added to reduce small sample biases. Consider two columns i and j from two independent profiles, which correspondingly have base frequency vectors f_bi _or f_bj_, and observed count vectors n_bi _or n_bj_. The ALLR statistic is formulated as:

ALLR=∑b=A..Tnbjln⁡(fbi/pb)+∑b=A..Tnbiln⁡(fbj/pb)∑b=A..Tnbi+nbj
 MathType@MTEF@5@5@+=feaafiart1ev1aaatCvAUfKttLearuWrP9MDH5MBPbIqV92AaeXatLxBI9gBaebbnrfifHhDYfgasaacH8akY=wiFfYdH8Gipec8Eeeu0xXdbba9frFj0=OqFfea0dXdd9vqai=hGuQ8kuc9pgc9s8qqaq=dirpe0xb9q8qiLsFr0=vr0=vr0dc8meaabaqaciaacaGaaeqabaqabeGadaaakeaacqWGbbqqcqWGmbatcqWGmbatcqWGsbGucqGH9aqpdaWcaaqaamaaqafabaGaemOBa42aaSbaaSqaaiabdkgaIjabdQgaQbqabaGccyGGSbaBcqGGUbGBcqGGOaakcqWGMbGzdaWgaaWcbaGaemOyaiMaemyAaKgabeaakiabc+caViabdchaWnaaBaaaleaacqWGIbGyaeqaaOGaeiykaKIaey4kaSYaaabuaeaacqWGUbGBdaWgaaWcbaGaemOyaiMaemyAaKgabeaakiGbcYgaSjabc6gaUjabcIcaOiabdAgaMnaaBaaaleaacqWGIbGycqWGQbGAaeqaaOGaei4la8IaemiCaa3aaSbaaSqaaiabdkgaIbqabaGccqGGPaqkaSqaaiabdkgaIjabg2da9iabdgeabjabc6caUiabc6caUiabdsfaubqab0GaeyyeIuoaaSqaaiabdkgaIjabg2da9iabdgeabjabc6caUiabc6caUiabdsfaubqab0GaeyyeIuoaaOqaamaaqafabaGaemOBa42aaSbaaSqaaiabdkgaIjabdMgaPbqabaGccqGHRaWkcqWGUbGBdaWgaaWcbaGaemOyaiMaemOAaOgabeaaaeaacqWGIbGycqGH9aqpcqWGbbqqcqGGUaGlcqGGUaGlcqWGubavaeqaniabggHiLdaaaaaa@776F@

The ALLR score between two aligned profiles is the sum of the scores between each pair of aligned positions. The expected score is negative, which makes this statistic suitable for Smith/Waterman alignment methods.

#### The TOLLR statistic

Datasets coming from experiments are often "corrupted samples," in which only a subset of the sequences contains the desired motif. For example, chromatin IP experiments may contain both sequences that are directly bound by the assayed transcription factor and sequences that are bound by another protein that happens to interact with the first protein. Motif discovery algorithms must define the correct motif based on sequences containing the true positive sites without incorporating sequences that don't contain true sites.

PhyloCon uses a greedy algorithm to compare profiles and to build new profiles in steps, or cycles. New profiles created in the current cycle always contain one more group of orthologous sequences than those generated from the previous cycle. We observe that by monitoring the trends of the best ALLR scores coming from comparisons in each cycle, we can discover the subset of sequences that contain true positives. Let's consider the scenario where, among a total of N orthologous groups, M groups share a conserved motif. The motif usually emerges in a few cycles, after which the best ALLR score from each cycle, usually corresponding to the shared motif, slowly decreases as weaker matching profiles are compared and incorporated. At the end of cycle M-1, the best ALLR score remains high although is lower than the previous cycle, as the resulting profile likely contains all true positives. However, in the next cycle the best ALLR score drops significantly because at least one false positive is forced into the comparison. To pinpoint this boundary, we introduced a related statistic called TOLLR (Total Log Likelihood Ratio), which is defined as the product of the ALLR statistic and the total number of sites from all genomes and all orthologous groups that constitute the motif. Following the trend of best TOLLR scores in each cycle we observed that, unlike the ALLR's continuously decreasing behavior, the best TOLLR score increases first, often peaking at a later cycle, and then changes only slightly. The reason is that when a profile corresponding to a true motif emerges, usually the true positive sites/profiles that fit the model described by this profile will be recruited for creating a new profile, therefore the total log likelihood ratio increases as more true positives are brought into the model from the entire search space. Once there are no more true positives left and a false positive is forced into the model, the total log likelihood ratio drops. Therefore, the peak TOLLR usually indicates the identification of the best overall motif.

#### PhyloCon motif discovery implementation details

The PhyloCon algorithm was implemented as a C program. All analysis, including those using the real data and those using the randomized control data, was done using PhyloCon-v3b, with default parameter settings, except for the parameter s being set to 0.5. This parameter determines the stringency and length of the initial multiple sequence alignments within each orthologous group. The top 50 profiles from each orthologous group were recorded for subsequent comparison. At each comparison step, the ALLR statistic was used to rank the most similar profile pairs and determine if two profiles should be merged. A new profile was generated whenever two profiles were merged, and the TOLLR score was given to the new profile. The program terminated when no pair of profile comparison gave an ALLR score higher than the default threshold (5.0). Finally, profiles were reported in the rank of their TOLLR scores.

#### Assessing motif significance

We used the approach of Harbison *et al*. to empirically estimate the significance level of the motif generated by PhyloCon and Converge. The number of promoters bound by transcription factors in the experiments ranges from 4 to 176, with an average of 55. From all promoters in the yeast genome where an orthologous sequence group could be formed based on sequences of multiple genomes, we randomly created datasets from 4 to 160 orthologous groups in size. For each sample size, 50 to 100 datasets were generated. Then we applied PhyloCon and Converge to these randomized datasets and estimated normal distributions for the motif scores (ALLR, TOLLR, and hypergeometric enrichment) at each sample size. After motif discovery on real datasets, motif scores were compared to the normal distribution of the most closely matching random sequence sample size. P-values were determined using z-scores calculated from the mean and standard deviation of this distribution.

### Combination of PhyloCon and converge motifs

The motifs produced by PhyloCon and Converge were assembled into a common catalogue of factor specificities. The set of motifs significant at p < 0.001 produced by each program were compiled and ranked by statistical significance. The motifs generated by each program were compared, with matches defined as an average Euclidean distance between the PSSM columns of less than 0.18. This empirical threshhold identified reasonable matches when the sequence logos of the motifs were compared visually.

In cases where either PhyloCon or Converge generated two or more instances of the same significant motifs we chose the one with the lowest p-value. When PhyloCon and Converge both found the same motif, and it was determined to be significant by both programs, we averaged the motifs. Averaging was performed, as in Harbison *et al*., by identifying the alignment of the motif matrices with minimum KL divergence (enforcing a minimum overlap of 6 bases), and then averaging the probabilities at each position. If there were no significant motifs common to both programs, the most statistically significant motif at a level of p < 0.001 was reported. This is the same strict significance criterion employed by Harbison and co-workers.

### Measurement of error rates

Estimates of false positive, true positive, false negative, and true negative rates for Converge, PhyloCon, and the combined set of motifs were calculated using the set of factors for which a specificity had been previously reported in the literature (see Additional file 1). A program was judged to produce a false positive when its top-ranked significant motif did not match the known specificity. When a matrix was available for the known specificity, a match was defined as an average Euclidean distance between the PSSM columns of < 0.18. For the remaining motifs, a match was determined empirically by assessing whether the motif PSSM was consistent with reported binding sites. True positives were defined as top-ranked statistically significant motifs that matched the known specificity. A false negative was defined as the case when the program produced no statistically significant motif, but the correct specificity was discovered by another program (PhyloCon, Converge, or one of the six programs from [3]). A true negative was defined as the case when the program produced no significant motif, and no other program was able to discover the known specificity.

### Regeneration of the yeast regulatory code

The yeast regulatory code was generated using the new catalogue of motifs and the methods described in [3]. For the purposes of generating the code, any motifs in the catalogue disagreeing with known specificities (false positives) were replaced with the literature motif. Any previously reported motifs that were not found by PhyloCon or Converge were added to the catalogue so that the regenerated map would be comprehensive. The *S. cerevisiae *genome was scanned for occurrences of the motifs in the catalogue using the same conservative criteria used by Harbison *et al*. A threshold cutoff of 60% of the maximum possible log-likelihood score for the motif defined a match. Only sites that were conserved in 3 out of the 4 yeast species, and corresponded to a probe bound by the factor at p < 0.001, were included in the regulatory code. We include a site in the map if it contains the factor's motif, it is conserved across at least 3 out of 4 yeast *sensu stricto *species, and it is bound at p < 0.001 in the location analysis of Harbison *et al*. A factor is said to have a regulatory interaction with a gene if there are one or more bound instances for that factor in the intergenic region upstream of a given gene. Other versions of the map that were generated with looser criteria for binding and/or conservation are available from the authors' website [11].

## Availability and requirements

PhyloCon is implemented as a C program on a Linux operating system. It is freely available for academic users. Non-academic users may require a license from Washington University. Converge is implemented in Python v. 2.2, with computationally intensive subroutines implemented in C++, on a Linux operating system. Both programs may be downloaded from [11].

## Authors' contributions

KDM and TW designed and implemented the algorithms, analyzed the data and drafted the manuscript. DBG participated in the statistical analysis. DKG provided technical insights and suggestions. GDS and EF conceived the study, participated in its design and coordination and helped to draft the manuscript. All authors read and approved the final manuscript.

## Supplementary Material

Additional File 1 Saccharomyces cerevisiae transcription factors with known DNA-binding specificities Click here for file

Additional File 2 Factor Specificities in the New Yeast Regulatory Map Click here for file

Additional File 3 Performance comparison between PhyloCon, Converge and the tree sampler implemented in Li and Wong. Click here for file

Additional File 4 Details of the Converge Algorithm. Click here for file

## Figures and Tables

**Table 1:** Transcription factor network motifs in the old and new regulatory codes

**Regulatory motif type**	**This study**	**Harbison *et al*.**
Autoregulation	16	12
Multi-component loop	15	5
Feed-forward loop	71	55
Single-input motif	91	72
Multi-input motif	481	392
Regulatory chain	1452	168
