# Supplementary material for: An improved map of conserved regulatory sites for Saccharomyces cerevisiae
Source: BMC Bioinformatics. 2006 Mar 7;7:113. doi: 10.1186/1471-2105-7-113 (PMC1435934; doi:10.1186/1471-2105-7-113)
Supplement: Additional File 1 — Saccharomyces cerevisiae transcription factors with known DNA-binding specificities [file 1471-2105-7-113-S1.doc]

**Additional file 1 – *Saccharomyces cerevisiae* transcription factors with known DNA-binding specificities**

| **REGULATOR** | **KNOWN SPECIFICITY** | **SOURCE^1^** | **REFERENCE** |
| --- | --- | --- | --- |
| ABF1 | RTCAYTNNNNACGW | Tfac |  |
| ACE2 | GCTGGT | SCPD |  |
| ADR1 | GGRGK | SCPD |  |
| AFT2 | ...AAAGTGCACCCATT… | YPD | [1,2] |
| ASH1 | YTGACT | YPD | [3] |
| AZF1 | TTTTTCTT | YPD | [4] |
| BAS1 | TGACTC | SCPD |  |
| CAD1 | TTACTAA | YPD | [5] |
| CBF1 | RTCACRTGA | SCPD |  |
| CIN5 | TTACTAA | YPD | [5] |
| CRZ1 | GWGGCTG |  | [6] |
| DAL80 | GATAA | YPD | [7] |
| DAL81 | AAAAGCCGCGGGCGGGATT | Tfac |  |
| DAL82 | GAAAATTGCGTT |  | [8] |
| ECM22 | CTCGTATAAGC |  | [9] |
| FKH1 | GGTAAACAA | Tfac |  |
| FKH2 | GGTAAACAA | Tfac |  |
| GAL4 | CGGNNNNNNNNNNNCCG | SCPD |  |
| GAL80 | CGGNNNNNNNNNNNCCG | SCPD |  |
| GAT1 | GATAA | YPD | [10] |
| GCN4 | ARTGACTCW | Tfac |  |
| GCR1 | GGCTTCCWC | Tfac |  |
| GLN3 | GATAAGATAAG | YPD | [11] |
| GZF3 | GATAAG | YPD | [11] |
| HAC1 | KGMCAGCGTGTC | Tfac |  |
| HAP1 | CGGNNNTANCGG | SCPD |  |
| HAP2 | CCAAT | YPD | [12] |
| HAP3 | CCAAT | YPD | [12] |
| HAP4 | YCNNCCAATNANM | Tfac |  |
| HAP5 | CCAAT | YPD | [12] |
| HSF1 | TTCTAGAANNTTCT | Tfac |  |
| INO2 | ATTTCACATC | Tfac |  |
| INO4 | CATGTGAAAT | YPD | [13] |
| LEU3 | YGCCGGTACCGGYK | SCPD |  |
| MAC1 | GAGCAAA | SCPD |  |
| MATA1 | ACATCA |  | [14] |
| MBP1 | ACGCGT | YPD | [15] |
| MCM1 | WTTCCYAAWNNGGTAA | Tfac |  |
| MET28 | TCACGTG |  | [16] |
| MET31 | AAACTGTGG | Tfac |  |
| MET32 | AAACTGTGG | Tfac |  |
| MET4 | AAACTGTGG |  | [17] |
| MIG1 | WWWWSYGGGG |  | [18] |
| MOT3 | YAGGYA | Tfac |  |
| MSN2 | MAGGGG | Tfac |  |
| MSN4 | MAGGGG | Tfac |  |
| NRG1 | CCCT |  | [19] |
| PDR1 | CCGCGG |  | [20] |
| PDR3 | TCCGCGGA | Tfac |  |
| PHO2 | ATTA |  | [21] |
| PHO4 | CACGTKNG | Tfac |  |
| PUT3 | CGGNNNNNNNNNNCCG | SCPD |  |
| RAP1 | WRMACCCATACAYY | Tfac |  |
| RCS1 | AAMTGGGTGCAKT | Tfac |  |
| RDS1 | CGGCCG |  |  |
| REB1 | TTACCCGG | Tfac |  |
| RGT1 | CGGANNA | YPD | [22] |
| RIM101 | TGCCAAG |  | [23] |
| RLM1 | CTAWWWWTAG |  | [24] |
| ROX1 | YSYATTGTT | Tfac |  |
| RPH1 | CCCCTTAAGG |  | [25] |
| RPN4 | GGTGGCAAA | Tfac |  |
| RTG1 | GGTCAC |  | [26] |
| RTG3 | GGTCAC |  | [26] |
| SIP4 | YCGGAYRRAWGG | SCPD |  |
| SKN7 | ATTTGGCYGGSCC | YPD | [27] |
| SKO1 | ACGTCA | SCPD |  |
| SMP1 | ACTACTAWWWWTAG | Tfac |  |
| STB5 | CGG |  | [28] |
| STE12 | ATGAAAC | Tfac |  |
| STP1 | RCGGCNNNRCGGC | YPD | [29] |
| SUM1 | AGYGWCACAAAAK | YPD | [30] |
| SUT1 | CGCG |  | [31] |
| SWI4 | CNCGAAA | SCPD |  |
| SWI5 | KGCTGR | SCPD |  |
| SWI6 | CNCGAAA | SCPD |  |
| TEC1 | CATTCY | YPD | [32] |
| TYE7 | CANNTG | YPD | [33] |
| UGA3 | CCGNNNNCGG | SCPD |  |
| UME6 | WGCCGCCGW | Tfac |  |
| XBP1 | CTTCGAG | Tfac |  |
| YAP1 | TTASTMA |  | [34] |
| YAP3 | TTACTAA | YPD | [5] |
| YAP6 | TTACTAA | YPD | [5] |
| YAP7 | TTACTAA | YPD | [5] |
| YHP1 | TAATTG |  | [35] |
| YOX1 | YAATA | YPD | [36] |
| ZAP1 | ACCCTAAAGGT | Tfac |  |

^1^SCPD: The Promoter Database of *Saccharomyces cerevisiae* , Tfac: TRANSFAC database, YPD: YPD database

**Supplemental Table 1 Bibliography**

1. Rutherford JC, Jaron S, Ray E, Brown PO, Winge DR (2001) A second iron-regulatory system in yeast independent of Aft1p. Proc Natl Acad Sci U S A 98: 14322-14327.

2. Rutherford JC, Jaron S, Winge DR (2003) Aft1p and Aft2p mediate iron-responsive gene expression in yeast through related promoter elements. J Biol Chem 278: 27636-27643.

3. Maxon ME, Herskowitz I (2001) Ash1p is a site-specific DNA-binding protein that actively represses transcription. Proc Natl Acad Sci U S A 98: 1495-1500.

4. Newcomb LL, Hall DD, Heideman W (2002) AZF1 is a glucose-dependent positive regulator of CLN3 transcription in Saccharomyces cerevisiae. Mol Cell Biol 22: 1607-1614.

5. Fernandes L, Rodrigues-Pousada C, Struhl K (1997) Yap, a novel family of eight bZIP proteins in Saccharomyces cerevisiae with distinct biological functions. Mol Cell Biol 17: 6982-6993.

6. Stathopoulos AM, Cyert MS (1997) Calcineurin acts through the CRZ1/TCN1-encoded transcription factor to regulate gene expression in yeast. Genes Dev 11: 3432-3444.

7. Rai R, Daugherty JR, Cooper TG (1995) UASNTR functioning in combination with other UAS elements underlies exceptional patterns of nitrogen regulation in Saccharomyces cerevisiae. Yeast 11: 247-260.

8. Dorrington RA, Cooper TG (1993) The DAL82 protein of Saccharomyces cerevisiae binds to the DAL upstream induction sequence (UIS). Nucleic Acids Res 21: 3777-3784.

9. Vik A, Rine J (2001) Upc2p and Ecm22p, dual regulators of sterol biosynthesis in Saccharomyces cerevisiae. Mol Cell Biol 21: 6395-6405.

10. van der Merwe GK, van Vuuren HJ, Cooper TG (2001) Cis-acting sites contributing to expression of divergently transcribed DAL1 and DAL4 genes in S. cerevisiae: a word of caution when correlating cis-acting sequences with genome-wide expression analyses. Curr Genet 39: 156-165.

11. Rowen DW, Esiobu N, Magasanik B (1997) Role of GATA factor Nil2p in nitrogen regulation of gene expression in Saccharomyces cerevisiae. J Bacteriol 179: 3761-3766.

12. McNabb DS, Xing Y, Guarente L (1995) Cloning of yeast HAP5: a novel subunit of a heterotrimeric complex required for CCAAT binding. Genes Dev 9: 47-58.

13. Bachhawat N, Ouyang Q, Henry SA (1995) Functional characterization of an inositol-sensitive upstream activation sequence in yeast. A cis-regulatory element responsible for inositol-choline mediated regulation of phospholipid biosynthesis. J Biol Chem 270: 25087-25095.

14. Jin Y, Zhong H, Vershon AK (1999) The yeast a1 and alpha2 homeodomain proteins do not contribute equally to heterodimeric DNA binding. Mol Cell Biol 19: 585-593.

15. Verma R, Patapoutian A, Gordon CB, Campbell JL (1991) Identification and purification of a factor that binds to the Mlu I cell cycle box of yeast DNA replication genes. Proc Natl Acad Sci U S A 88: 7155-7159.

16. Kuras L, Barbey R, Thomas D (1997) Assembly of a bZIP-bHLH transcription activation complex: formation of the yeast Cbf1-Met4-Met28 complex is regulated through Met28 stimulation of Cbf1 DNA binding. Embo J 16: 2441-2451.

17. Blaiseau PL, Thomas D (1998) Multiple transcriptional activation complexes tether the yeast activator Met4 to DNA. Embo J 17: 6327-6336.

18. Lutfiyya LL, Iyer VR, DeRisi J, DeVit MJ, Brown PO, et al. (1998) Characterization of three related glucose repressors and genes they regulate in Saccharomyces cerevisiae. Genetics 150: 1377-1391.

19. Park SH, Koh SS, Chun JH, Hwang HJ, Kang HS (1999) Nrg1 is a transcriptional repressor for glucose repression of STA1 gene expression in Saccharomyces cerevisiae. Mol Cell Biol 19: 2044-2050.

20. Hellauer K, Akache B, MacPherson S, Sirard E, Turcotte B (2002) Zinc cluster protein Rdr1p is a transcriptional repressor of the PDR5 gene encoding a multidrug transporter. J Biol Chem 277: 17671-17676.

21. Barbaric S, Munsterkotter M, Svaren J, Horz W (1996) The homeodomain protein Pho2 and the basic-helix-loop-helix protein Pho4 bind DNA cooperatively at the yeast PHO5 promoter. Nucleic Acids Res 24: 4479-4486.

22. Kim JH, Polish J, Johnston M (2003) Specificity and regulation of DNA binding by the yeast glucose transporter gene repressor Rgt1. Mol Cell Biol 23: 5208-5216.

23. Lamb TM, Mitchell AP (2003) The transcription factor Rim101p governs ion tolerance and cell differentiation by direct repression of the regulatory genes NRG1 and SMP1 in Saccharomyces cerevisiae. Mol Cell Biol 23: 677-686.

24. Jung US, Levin DE (1999) Genome-wide analysis of gene expression regulated by the yeast cell wall integrity signalling pathway. Mol Microbiol 34: 1049-1057.

25. Jang YK, Wang L, Sancar GB (1999) RPH1 and GIS1 are damage-responsive repressors of PHR1. Mol Cell Biol 19: 7630-7638.

26. Jia Y, Rothermel B, Thornton J, Butow RA (1997) A basic helix-loop-helix-leucine zipper transcription complex in yeast functions in a signaling pathway from mitochondria to the nucleus. Mol Cell Biol 17: 1110-1117.

27. Li S, Dean S, Li Z, Horecka J, Deschenes RJ, et al. (2002) The eukaryotic two-component histidine kinase Sln1p regulates OCH1 via the transcription factor, Skn7p. Mol Biol Cell 13: 412-424.

28. Akache B, MacPherson S, Sylvain MA, Turcotte B (2004) Complex interplay among regulators of drug resistance genes in Saccharomyces cerevisiae. J Biol Chem 279: 27855-27860.

29. Nielsen PS, van den Hazel B, Didion T, de Boer M, Jorgensen M, et al. (2001) Transcriptional regulation of the Saccharomyces cerevisiae amino acid permease gene BAP2. Mol Gen Genet 264: 613-622.

30. Pierce M, Benjamin KR, Montano SP, Georgiadis MM, Winter E, et al. (2003) Sum1 and Ndt80 proteins compete for binding to middle sporulation element sequences that control meiotic gene expression. Mol Cell Biol 23: 4814-4825.

31. Regnacq M, Alimardani P, El Moudni B, Berges T (2001) SUT1p interaction with Cyc8p(Ssn6p) relieves hypoxic genes from Cyc8p-Tup1p repression in Saccharomyces cerevisiae. Mol Microbiol 40: 1085-1096.

32. Madhani HD, Fink GR (1997) Combinatorial control required for the specificity of yeast MAPK signaling. Science 275: 1314-1317.

33. Sato T, Lopez MC, Sugioka S, Jigami Y, Baker HV, et al. (1999) The E-box DNA binding protein Sgc1p suppresses the gcr2 mutation, which is involved in transcriptional activation of glycolytic genes in Saccharomyces cerevisiae. FEBS Lett 463: 307-311.

34. Nguyen DT, Alarco AM, Raymond M (2001) Multiple Yap1p-binding sites mediate induction of the yeast major facilitator FLR1 gene in response to drugs, oxidants, and alkylating agents. J Biol Chem 276: 1138-1145.

35. Kunoh T, Kaneko Y, Harashima S (2000) YHP1 encodes a new homeoprotein that binds to the IME1 promoter in Saccharomyces cerevisiae. Yeast 16: 439-449.

36. Pramila T, Miles S, GuhaThakurta D, Jemiolo D, Breeden LL (2002) Conserved homeodomain proteins interact with MADS box protein Mcm1 to restrict ECB-dependent transcription to the M/G1 phase of the cell cycle. Genes Dev 16: 3034-3045.
