# Supplementary material for: An improved map of conserved regulatory sites for Saccharomyces cerevisiae
Source: BMC Bioinformatics. 2006 Mar 7;7:113. doi: 10.1186/1471-2105-7-113 (PMC1435934; doi:10.1186/1471-2105-7-113)
Supplement: Additional File 2 — Factor Specificities in the New Yeast Regulatory Map [file 1471-2105-7-113-S2.doc]

**Additional File 2 - Factor Specificities in the New Yeast Regulatory Map**

| **Factor** | **Reported Specificity** | **Literature Specificity** | **Program^1^** |
| --- | --- | --- | --- |
| ABF1 | .rTCAyt.y..ACG. | RTCAYTNNNNACGW | c |
| ACE2 | arCCmgcm | ACCAGC | a |
| ADR1 | .GGrGk | GGRGK | d |
| AFT2 | kgCACCc | ...AAAGTGCACCCATT… | c |
| ARG80 | wGACkC |  | a |
| ARG81 | .TGACTCy |  | b |
| ARO80 | CCG.gr.TwrCCGmsAkTTGCCG |  | b |
| ARR1 | A.yTrAAt |  | a |
| ASH1 | cCr.RTcrGG | ATCAR | a |
| AZF1 | YwTTkcKkTyyckgykky | TTTTTCTT | d |
| BAS1 | TGACTCy... | TGACTC | c |
| CAD1 | gcTkAcTAAT | TTACTAA | c |
| CBF1 | GTCACGTG | RTCACRTGA | c |
| CHA4 | tGCGAtgar |  | b |
| CIN5 | mtTAcrTAA | TTACTAA | c |
| CRZ1 | GwGGCTG | GwGGCTG | d |
| CST6 | tgCATTT. |  | b |
| DAL80 | GATAA | GATAA | d |
| DAL81 | AAAAGCCGCGGGCGGGATT | AAAAGCCGCGGGCGGGATT | d |
| DAL82 | AAa.wTgyG. | GAAAATTGCGTT | a |
| DIG1 | .........tGAAAc. |  | c |
| ECM22 | CTCGTATAAGC | CTCGTATAAGC | d |
| FHL1 | rTGTAcGGrT |  | c |
| FKH1 | ....gTAAACAa | GGTAAACAA | c |
| FKH2 | ...a...gTAAACAa | GGTAAACAA | c |
| GAL4 | CGGas.rsw.y.s.CCGa | CGGNNNNNNNNNNNCCG | c |
| GAL80 | CGG...........CCG | CGGNNNNNNNNNNNCCG | d |
| GAT1 | aGATAAG | GATAA | d |
| GAT3 | ATaacATG |  | a |
| GCN4 | rTGACTca | ARTGACTCW | c |
| GCR1 | .ggCTTCCw | GGCTTCCWC | b |
| GCR2 | gCTTCCw |  | b |
| GLN3 | mGATaAgrta | GATAAGATAAG | a |
| GTS1 | TAcCAA |  | a |
| GZF3 | GATAAG | GATAA | d |
| HAC1 | AkGmCACGTA | KGMCAGCGTGTC | a |
| HAP1 | CGatAa..sC | CGGNNNTANCGG | a |
| HAP2 | CCAAT.a. | CCAAT | a |
| HAP3 | CCAAT | CCAAT | d |
| HAP4 | .CCAATcA....... | YCNNCCAATNANM | c |
| HAP5 | CCAAT | CCAAT | d |
| HSF1 | rGAA..TtctrGAA | AGAANNTTCTAGAA | c |
| IME1 | CSGCsGAG |  | a |
| INO2 | gCATGTGAA | GATGTGAAAT | c |
| INO4 | gCATGTGAA | CATGTGAAAT | c |
| IXR1 | AArcmrgRAGCGGkG |  | a |
| LEU3 | CCGGtmCCGG | YGCCGGTACCGGYK | c |
| MAC1 | Rc.AcggTaA | GAGCAAA | a |
| MATA1 | ACATCA | ACATCA | d |
| MBP1 | aCGCGTc | ACGCGT | c |
| MCM1 | tktCC..wTt.GGAAA | WTTCCYAAWNNGGTAA | c |
| MET28 | TCACGTG | TCACGTG | d |
| MET31 | .gTGTGk. | AAACTGTGG | b |
| MET32 | mArcTGTGGC | AAACTGTGG | b |
| MET4 | AayTGTGG |  | a |
| MIG1 | wwwwsyGGGG | WWWWSYGGGG | d |
| MOT3 | yAGGyA | YAGGYA | d |
| MSN2 | mAGGGG. | MAGGGG | b |
| MSN4 | aAGGGG. | MAGGGG | b |
| NDD1 | rAargGsAAA |  | a |
| NRG1 | ggACCCt | CCCT | c |
| OPI1 | .GAACC. | CGAACCR | b |
| PDR1 | ccGCCgRAwra | CCGCGG | d |
| PDR3 | TCCGCGGA | TCCGCGGA | d |
| PHD1 | RGGcAm |  | a |
| PHO2 | AYTAAr | ATTA | a |
| PHO4 | CACGTG. | CACGTKNG | c |
| PUT3 | CGGGAAGCCM...c.. | CGGNNNNNNNNNNCCG | c |
| RAP1 | ..ACCCR.aCMy | WRMACCCATACAYY | c |
| RCS1 | rGGTGca | AAMTGGGTGCAKT | c |
| RDS1 | CGGCCG | CGGCCG | a |
| REB1 | TTACCCGs | TTACCCGG | c |
| RFX1 | GTtgyCATgG.aAc |  | c |
| RGT1 | syCGGAAAAA | CGGANNA | b |
| RIM101 | TGCCAAG | TGCCAAG | d |
| RLM1 | TATTTatAga | CTAWWWWTAG | a |
| RLR1 | ACAGTwyTtwcAGkw |  | a |
| RME1 | Y..AaAGGaa |  | a |
| ROX1 | arMAsCcCgs | YNNYYACCCG | a |
| RPH1 | CCCCTTAAGG | CCCCTTAAGG | d |
| RPN4 | .GGTGGCaAaw | GGTGGCAAA | c |
| RTG3 | rTGACkC. | GGTCAC | b |
| SFL1 | GAAGCTTC | GAAGCTTC | d |
| SFP1 | rTGTAyGGrT |  | c |
| SIP4 | .tCGG.YsWATGGRr | YCGGAYRRAWGG | a |
| SKN7 | gGscyrGm | ATTTGGCYGGSCC | a |
| SKO1 | yACGTCAt | ACGTCA | c |
| SMP1 | ACTACTAwwwwTAG | ACTACTAWWWWTAG | d |
| SNF1 | CCGrGrATCGAACyCGG |  | b |
| SNT2 | .tGrTAGCGCCr... |  | c |
| SOK2 | .cAGGmAm |  | a |
| SPT2 | w.TTRamkAR |  | a |
| SPT23 | rAAATsaA |  | d |
| STB1 | CGCGAAAa |  | c |
| STB2 | CGSGTAA. |  | b |
| STB4 | TCGg..CGA |  | d |
| STB5 | CGGtstTata | CGG | c |
| STE12 | tGAAACa | ATGAAAC | c |
| STP1 | yGCGGCk. | RCGGCNNNRCGGC | b |
| STP4 | CGCCGYKKsRSrCGM |  | a |
| SUM1 | ...yGwCAswAA.. | AGYGWCACAAAAK | c |
| SUT1 | .gCsGgg | CGCG | c |
| SWI4 | .CrCGAAA. | CNCGAAA | c |
| SWI5 | tGCTGg.. | KGCTGR | c |
| SWI6 | CGCGTy | CNCGAAA | a |
| TEC1 | .rCATTCy | CATTCY | b |
| THI2 | .GmAACyswwAGArCy |  | c |
| TYE7 | .tcaCGTG | CANNTG | c |
| UGA3 | CCG....CGG | CCGNNNNCGG | d |
| UME6 | tAGCCGCcsa | WGCCGCCGW | c |
| XBP1 | cTCGAGG. | CTTCGAG | b |
| YAP1 | .gcTgAcTAA. | TTASTMA | c |
| YAP3 | TTACTAA | TTACTAA | d |
| YAP5 | ARrCAT |  | a |
| YAP6 | tTACrTaa | TTACTAA | b |
| YAP7 | .mTkAcTAAt | TTACTAA | c |
| YDR026C | ACCCGG |  | a |
| YDR520C | tCtCCGGCGga |  | c |
| YER051W | TTCGAA |  | a |
| YHP1 | TAATTG | TAATTG | d |
| YML081W | CCAGTcTKAM |  | a |
| YOX1 | mATTASgTTTCCYRAwmgGG | YAATTA | b |
| YRR1 | TttTGTTACSCr |  | b |
| ZAP1 | ACCYTmArGGT.rTG | ACCCTAAAGGT | b |

a: specificity discovered by Converge, b: specificity discovered by PhyloCon, c: specificity discovered by both programs, d: specificity added from literature source or Harbison et al.
