# Supplementary material for: An improved map of conserved regulatory sites for Saccharomyces cerevisiae
Source: BMC Bioinformatics. 2006 Mar 7;7:113. doi: 10.1186/1471-2105-7-113 (PMC1435934; doi:10.1186/1471-2105-7-113)
Supplement: Additional File 3 — Performance comparison between PhyloCon, Converge and the tree sampler implemented in Li and Wong. [file 1471-2105-7-113-S3.doc]

**Additional File 3 – Performance comparison between PhyloCon, Converge and the tree sampler implemented in Li and Wong [1].**

Table Legend: Column 1 contains names of transcription factors. Column 2 contains a text representation of the known specificity for each protein using IUPAC codes; Columns 3 to 5 contain the motifs discovered by Li and Wong, PhyloCon and Converge. If a motif matches the known specificity, the corresponding region is underlined. If it doesn’t match the known specificity, the motif is indicated in red italics. At the end of the table, the accuracy of each program is reported at the base-pair and motif level.

| **Factor** | **Literature** | **Li and Wong** | **PhyloCon^a^** | **Converge^a^** |
| --- | --- | --- | --- | --- |
| Abf1 | rTCAyTnnnnACGw | TCACTTTATACGAA | naTCAcTwtnnACGa | YCGTRYRWARTGAYA |
| Ace2 | GCTGGT | TGCTGGCCCA | *wAAACAAAc* | GCwGG. |
| Azf1 | AAGAAAAA | AAAAAGAAAAAAAA | TTTTCTT^a^ | *y.YGaRmgywrk.wAYgSGa* |
| Bas1 | TGACTC | GACTCTGCCTAA | nTGACTCy | wwtGAsTC.t..Y.. |
| Cad1 | TTACTAA | TTACTGAAAAAAA | gcTtAcTAAT | ATTAgTMAgC |
| Cbf1 | rTCACrTGA | GGTCACGTGGTCA | GTCACGTGn | kCACGTGm |
| Cin5 | TTACRTAA | *NULL* | cTTACaTAAk | aTtAcrTAAg..... |
| Fkh1 | GGTAAACAA | AAAGATAAACAAA | GGTAAACAAS | raa.gtAAACAa... |
| Fkh2 | GGTAAACAA | GCGGGTAAAT | GGTAAACAA | gg.aAaa.gTAAAcA |
| Gal4 | CGGnnnnnnnnnnnCCG | TTCGGAACAAGGCGGACC | CGGastasanTgcnCCG | c.CGGas.rcW.yss.CCGA |
| Gcn4 | ArTGACTCw | TGACTCAT | aTGACTCAn | TGAsTCAy |
| Gcr1 | GGCTTCCwC | GTGTGTGGCTTCC | rgGCTTCcw | *AAcytyTT* |
| Gln3 | GATAAGATAAG | GAGAGATAAAA | wGATAAg^a^ | wGATaA |
| Hac1 | kGmCAGCGTGTC | *TGCCACGTAA* | *CTGAAAA* | AkGmCACGTA |
| Hap4 | CCAAT | TTTCTTCCAAT | aCCAATcAr | .ycT.ATtGsy.cr. |
| Hap5 | CCAAT | TTTTTCCAAT | rCCAAt^a^ | rGCcAA^a^ |
| Hsf1 | AGAAnnTTCTAGAA | *NULL* | RgAAtnTTCTAGAA | .TTCya.ra..TTCy |
| Ino2 | GATGTGAAAT | AGCATGTGAAAAC | gCATGTGAAr | .gCATGtGaA |
| Ino4 | GATGTGAAAT | CATGTGAAAAT | rnCATGTGAA | gCATGTGAAa |
| Leu3 | yGCCGGTACCGGyk | CGGAACCGGAAGA | gkCCGGTaCCGG | ccggtaccgg |
| Mac1 | GAGCAAA | TGAGCAAAAAAAA | GcGCAAttTw | GsGCAA |
| Mbp1 | ACGCGT | AACGCGTCACGA | ACGCGTC | rACGCGt |
| Mcm1 | wTTCCyAAwnnGGTAA | CCAAAATAGGAAAT | TTTCCyaAtnrGGaaA | .rTTtCC.rAw..GGamA.. |
| Met31 | AAACTGTGG | TGTGGCGTA | ngTGTGgy | ATTGTG^a^ |
| Met32 | AAACTGTGG | *AGCACGAGAAAAAA* | aAacTGTGGC | *CACGTGA.rygrs* |
| Met4 | AAACTGTGG | CTGTGGCAA | *TCACGTGAG* | cAr.TGTGGC |
| Mot3 | yAGGyA | GAAGGGAAA | KGATGAGGYAA^a^ | *SYKCGRYMMMRARSG* |
| Msn4 | mAGGGG | CCCCTGAAAA | aAGGGGn | *AagAr...s.GCGrr* |
| Nrg1 | GGaCCCT | CCCCTCCTCT | ngGACCCtk | ggaCCCT |
| Pdr1 | CCGCGG | *CACACCCACACACC* | *SGAAAAA* | *r.Tryr.rGg* |
| Pdr3 | TCCGCGGA | *NULL* | *NULL* | *NULL* |
| Pho4 | cacgtkng | *TCTTTCTTG* | gCACGTGGga | CACGTSgs |
| Rap1 | wrmACCCATACAyy | ACACCCATACATCT | aCACCCANaCac | CAyCCrtrCAyc |
| Rcs1 | AmTGCACCCA | *TTTTTCAACTT* | TGCACCC | cACcc....yrr |
| Reb1 | TTACCCGG | CCGGGTAACAAAAA | TTACCCGS | gTTACCCGw |
| Rfx1^b^ | GTTGCCATGGCG | TCCGTTGCCATGGC | GTtGYCATGGyaay | GTTgycatgGcAACr |
| Rlm1 | CTAwwwwTAG | *TTTGCCGAG* | cTAaAAATAGa^a^ | TATTTatAga |
| Rox1 | ysyATTGTT, YNNYYACCCG | *GCGTGGGGTAA* | *AAGGGCG* | arMAsCcCgs |
| Rph1 | CCCCTTAAGG | *CGCGCGCTCAGGAG* | *RgGgRgGgGGcGGgGG* | *sCSSCCtScc* |
| Rpn4 | GGTGGCAAA | *CGGCTCACAAA* | cGGtGGCAAAA | GGTGGCAAA |
| Skn7 | GnCnnGsCs | GCGGCTGGCCA | rGGcCcaGcCCg^a^ | yCy.gsCc |
| Sko1 | ACGTCA | TTACGTCA | tACGTCATn | yAcGtCAT |
| Sip4 | yCGGAyrrAwGG | *ACCCGGAA* | CGGnTgAATGG^a^ | .tCGG.YsWATGGRr |
| Smp1 | ACTACTAwwwwTAG | *NULL* | *AGCCAGS* | *Aaaa.tAgaa* |
| Ste12 | ATGAAACA | TTTGAAACAA | nTGAAACa | t.Cyyraw..ggaaa |
| Sum1 | AGyGwCACAAAAk | AGTGTCACAAA | YGtCAgwAA | yGwCAswAAwkm |
| Sut1 | AACGCGCAGG, AACGCGTGCC, ATCGCGCAATT | GCGCGGAAAA | *GCGGGGS* | *.cCGsrsc* |
| Swi4 | CnCGAAA | ACGCGAAA | nCgCGAAA | ..ys...CgCG.aAa |
| Swi5 | TGCTGGT | GGTGCGGGTA | tGCTGg | tgCtggyt |
| Swi6 | CnCGAAA | ACGCGTCGCGA | ncaCGAAAn^a^ | rrCGCGwma |
| Ume6 | wGCCGCCGw | CCTCGGCGGCTAA | AGCCGCCGAG | wTaGCCGCcsa |
| Yap1 | TGAsTCAG,  TTAGTAA | GCCGCTACTAAA | TTaGTMAGc | cYkAcTAA |
| Zap1 | ACCCTAAAGGT | CCCTCAAGGTCAAA | ACCtTaAaGGTyaTg | RACCTTkArGgT^a^ |
|  |  |  |  |  |
| **Number**  **Correct** |  | **39** | **44** | **43** |
| **% Correct** |  | **74%** | **83%** | **81%** |
| **Total bases** | **493** | **564** | **499** | **575** |
| **Matching**  **bases** |  | **301** | **378** | **362** |
| **Sn**^c^ |  | **0.6105** | **0.7667** | **0.7343** |
| **Sp**^d^ |  | **0.5337** | **0.7575** | **0.6296** |

^a^ The top-ranked motifs produced by PhyloCon and Converge are included even if they did not meet the significance thresholds necessary for inclusion in the regulatory map.

^b^ The specificity for Rfx1 is not included in the list of known motifs in Table S.1.

^c^ Sensitivity (Sn) is defined as the number of correct bases divided by the total number of bases in the known motifs.

^d^ Specificity (Sp) is defined as the number of correct bases divided by the total number of bases in the predicted motifs.

1. Li X, Wong WH (2005) Sampling motifs on phylogenetic trees. Proc Natl Acad Sci U S A 102: 9481-9486 Epub 2005 Jun 9427.
