# Supplementary material for: An improved map of conserved regulatory sites for Saccharomyces cerevisiae
Source: BMC Bioinformatics. 2006 Mar 7;7:113. doi: 10.1186/1471-2105-7-113 (PMC1435934; doi:10.1186/1471-2105-7-113)
Supplement: Additional File 4 — Details of the Converge Algorithm. [file 1471-2105-7-113-S4.doc]

# Additional File 4 – The Converge Algorithm

Converge attempts to learn DNA sequence motifs present in the upstream regions of co-regulated or co-bound genes in the genome of a species of interest (i.e. a primary genome) by using conservation information in the form of pair-wise sequence alignments of phylogenetically related species. The Converge algorithm makes use of the fact that transcription factor binding motifs will tend to be conserved in orthologous regions of related species. Below we describe the algorithm in detail. A glossary of the terms used in the equations is provided at the end of this document.

**Overview**

A schematic diagram of the Converge workflow is shown below:

Figure 1 - Converge Workflow Diagram

The EM learning of motif sequences by Converge is preceded by seed selection step where initial starting points for EM are chosen. The sequences in the primary genome are scanned for statistically over-represented *w*-mers and the top twenty statistically overrepresented *w*-mers are used to initialize the PSSM for the first step of EM.

Expectation Maximization is run to convergence for each seed, and the resulting motif candidates are scored using an enrichment statistic to allow their statistical significance to be tested in a principled manner. The enrichment score is fit to a normal distribution generated using enrichment scores of randomized data runs for a similar number of sequence alignments and for the same motif size.

# Probabilistic Model

In the Converge framework, we start by specifying a motif width *w*. The observed data consists of a series of N primary genome sequences aligned (pair-wise) to orthologous sequence from supporting genomes, which we refer to as the vector **X**. Each set of pair-wise alignments is indexed over M possible *w*-mers and P genomes, and is assumed to contain either one or zero motifs in the primary genome as in the zero-or-one-occurrence-per-sequence (ZOOPS) model of Bailey and Elkan.

Regions of sequence are treated as arising from either a background distribution or a DNA motif distribution. The motif distribution is modeled using a position-specific scoring matrix (PSSM):

$$\Pi=\left[\pi_{1}...\pi_{w}\right]$$

Equation 1 – The position specific scoring matrix

Where each **π***_i_* is a multinomial distribution representing the expected frequency of each base at position *i* in the motif. For sequences flanking the motif region, the distribution is modeled as arising from a 4^th^ order Markov background, which in practice takes the from of a probability table with an entry for each possible 5-mer of base sequence, and which we denote by Π*_k_*, where *k* refers to the genome the background was calculated from. Converge assumes that the motif and background probabilities are independent.

The Converge framework attempts to model three important characteristics of the observed data: regions in the pairwise alignments that contain gaps should be treated differently than those without gaps, a given sequence may or may not contain the motif we are attempting to learn, and even when the motif is present in the primary genome it may not be present in the aligned supporting genomes. This treatment is made possible by the definition of two additional variables included in the data set. The gap indicator variables G*_i,j,k_* take the value of 1 if for alignment *i*, position *j*, genome *k*, a gap is present in the motif window of width *w* that begins at position *j*. We also define a second set of binary variables, the Z*_i,j,k_*, that indicate whether a functional motif is present in alignment *i*, at position *j*, in genome *k*.

Now two simplifying assumptions are introduced. First, it is assumed that a functional motif is only present in an aligned supporting genome if it also present at the corresponding position in the primary genome. If the primary genome is indexed by *k* = 1, this is equivalent to saying that, for all *k* = {*1…P*}, Z*_i,j,k_* is equal to zero with probability 1.0 if Z*_i,j,1_* is equal to zero. Second, it is assumed that given the value of Z*_i,j,k_*, the probability of observing the aligned sequence for genome *k* is independent of the other aligned sequences and the primary sequence. Now, the log probability of the data can be factored as follows:

$$\begin{matrix} \log P\left(\mathbf{X},\mathbf{G},\mathbf{Z}|\mathbf{\Psi}\right)=\log P\left(\mathbf{X}|\mathbf{Z},\mathbf{G},\mathbf{\Psi}\right)+\log P\left(\mathbf{G}|\mathbf{Z},\mathbf{\Psi}\right) \\ +\log P\left(\mathbf{Z}_{k\neq1}|\mathbf{Z}_{k=1},\mathbf{\Psi}\right)+\log P\left(\mathbf{Z}_{k=1}|\mathbf{\Psi}\right) \end{matrix}$$

Equation 2 – The Converge Probability Model

Here $\mathbf{Z}_{k=1}$ denotes the Z*_i,j,1_*’s for all *i* and *j*, $\mathbf{Z}_{k\neq1}$ denotes the Z*_i,j,k_*’s where *k*≠1, and **Ψ** denotes the parameters associated with the probability mass functions. Now we define each term in equation 2 as follows:

$$\begin{matrix} \log P\left(\mathbf{X}|\mathbf{Z},\mathbf{G},\mathbf{\Psi}\right)= \\ \sum_{i=1}^{N} \sum_{j=1}^{M} Z_{i,j,1}\sum_{k=1}^{P} \left\{\begin{matrix} P\left(window sequence|\mathbf{Z},G_{i,j,k},\mathbf{\Psi}^{window}\right) \\ +P\left(flank sequence|\mathbf{Z},\mathbf{\Psi}^{background}\right) \end{matrix}\right\} \\ +\sum_{i=1}^{N} \left(1-Q_{i}\right)P\left(background|\mathbf{\Psi}^{background}\right) \end{matrix}$$

Equation 3

Where,

$$Q_{i}=\sum_{j=1}^{M} Z_{i,j,1}$$

Equation 4

If there is no functional motif present in the primary sequence, the first term of equation 3 will be equal to zero and the conditional probability of the sequence will simply be the probability it was emitted by the background model. If there is a functional motif present in the primary sequence, one of the Z*_i,j,1_*’s will be non-zero and, assuming independence between the motif window and the flanking sequence, the log probability of the observed sequence is given by the sum of the window sequence log probability and the log probability of the flanking sequence. The expressions for the probability of the sequence in the motif window and the flanking sequence for alignment *i*, position *j*, and genome *k*, are modeled as shown below:

$$\begin{matrix} \log P\left(window sequence|\mathbf{Z},G_{i,j,k},\mathbf{\Psi}^{window}\right)= \\ \sum_{c=1}^{W} \left[\begin{matrix} G_{i,j,k}\left(Z_{i,j,k}\mathbf{\Pi}_{m}^{1}\left[c,X_{i,j+c,k}\right]+\left(1-Z_{i,j,k}\right)\mathbf{\Pi}_{bg,k}^{1}\left[X_{i,j+c,k}\right]\right)+ \\ \left(1-G_{i,j,k}\right)\left(Z_{i,j,k}\mathbf{\Pi}_{m}^{0}\left[c,X_{i,j+c,k}\right]+\left(1-Z_{i,j,k}\right)\mathbf{\Pi}_{bg,k}^{0}\left[X_{i,j+c,k}\right]\right) \end{matrix}\right] \end{matrix}$$

Equation 5

Equation 6

In equation 5 one of two probability models is selected depending on the value of the gap indicator variable. The value of the Z*_i,j,k_* selects either a motif model or a background model. When Z*_i,j,k_* is one, the probability of the sequence in the window is calculated using the appropriate PSSM indexed by position *c*, and base X_i,j+c,k_ ($\mathbf{\Pi}_{m}^{1}$for G_i,j,k_=1 or $\mathbf{\Pi}_{m}^{0}$ for G_i,j,k_=0), when its value is zero the probability is calculated using a 1^st^ order background table, indexed by base X_i,j+c,k_, for the appropriate genome *k* ($\mathbf{\Pi}_{bg,k}^{1}$for G_i,j,k_=1 or $\mathbf{\Pi}_{bg,k}^{0}$ for G_i,j,k_=0). Equation 5 assumes independence between positions in the motif window. Equation 6 shows that the probability of the sequence flanking the motif window is calculated using the 4^th^ order Markov background, indexed by $\tilde{X}_{i,c,k}$, the 5-tuple of sequence in the alignment beginning at position *c*, and genome *k*. In a similar fashion, the final term in equation 3 is defined as follows:

Equation 7

The second term of equation 2 models the probability of observing a gap in the motif window, given the value of the Z’s, and will in general be different for each aligned genome:

$$\log P\left(\mathbf{G}|\mathbf{Z},\mathbf{\Psi}\right)=\sum_{i=1}^{N} \sum_{j=1}^{M} Z_{i,j,1}\sum_{k=1}^{P} \left[\begin{matrix} Z_{i,j,k}\left(G_{i,j,k}\log\zeta_{k,1}+\left(1-G_{i,j,k}\right)\log\left(1-\zeta_{k,1}\right)\right) \\ +\left(1-Z_{i,j,k}\right)\left(G_{i,j,k}\log\zeta_{k,0}+\left(1-G_{i,j,k}\right)\log\left(1-\zeta_{k,0}\right)\right) \end{matrix}\right]$$

Equation 8

The G_i,j,k_’s are generated from two distinct binomial distributions selected by the value of the corresponding Z_i,j,k_. When Z_i,j,k_ is equal to one the value of G_i,j,k_ is generated from the binomial distribution with parameter $\zeta_{k,1}$, otherwise it is generated from a binomial distribution with parameter $\zeta_{k,0}$. This models our belief that the likelihood of observing a gap in an aligned sequence should be different depending on whether a functional motif is present in the primary sequence. The G_i,j,k_’s are also assumed to be independent across the different genomes.

The third term in equation 2 describes the probability of the Z_i,j,k_’s for k≠1 given the value of the Z_i,j,1_’s.

$$\log P\left(\mathbf{Z}_{k\neq1}|\mathbf{Z}_{k=1},\mathbf{\theta}\right)=\sum_{i=1}^{N} \sum_{j=1}^{M} Z_{i,j,1}\sum_{k=2}^{P} \left[Z_{i,j,k}\log\theta_{k}+\left(1-Z_{i,j,k}\right)\log\left(1-\theta_{k}\right)\right]$$

Equation 9

Since we constrain the Z_i,j,k_’s to be zero unless Z_i,j,1_ is zero, equation 9 models the probability of the Z_i,j,k_’s as arising from a single binomial distribution with parameter $\theta_{k}$ that represents the probability of observing a functional motif in the aligned genome *k*, given the presence of a functional motif in the primary genome.

The final term in the joint log probability distribution models the *a priori* probability of a functional motif being present at a particular alignment position in the primary genome:

$$\log P\left(\mathbf{Z}_{k=1}|\lambda\right)=\sum_{i=1}^{N} \sum_{j=1}^{M} Z_{i,j,1}\log\lambda+\sum_{i=1}^{N} \left(1-Q_{i}\right)\log\left(1-\gamma\right)$$

Equation 10

In equation 10, the parameter$\gamma$, is defined as the *a priori* probability of a motif being present in a given alignment, and the parameter $\lambda$ is defined as$\gamma/M$, or the *a priori* probability of a functional motif being present at any given position in the alignment.

**EM Algorithm**

Given the probability model outlined in the previous section, Converge attempts to learn the functional binding motifs present in the data set using the EM algorithm; an iterative coordinate ascent on the joint probability function of equation 2, that first calculates the expected value of the hidden variables Z, and then uses that expectation to re-estimate the values of the parameters **Π**, **ζ**, **θ**, and γ.

**E Step**:

In the E-step, Converge calculates the expected log likelihood of the data over the distribution of the hidden variables **Z**, which takes the form:

Equation 11

**M Step**:

Taking the partial derivative of equation 11 with respect to the parameters **Π**, **ζ**, **θ**, and λ, and setting the result equal to zero, we derive the M-step update equations for the parameters of our distribution:

$$\lambda^{(t+1)}=\frac{\sum_{i=1}^{N} \sum_{j=1}^{M} E\left[Z_{i,j,1}\right]}{NM}$$

Equation 12

$$\theta_{k}^{(t+1)}=\frac{\sum_{i=1}^{N} \sum_{j=1}^{M} E\left[Z_{i,j,k}\right]}{\sum_{i=1}^{N} \sum_{j=1}^{M} E\left[Z_{i,j,1}\right]}$$

Equation 13

$$\zeta_{k,1}^{(t+1)}=\frac{\sum_{i=1}^{N} \sum_{j=1}^{M} G_{i,j,k}E\left[Z_{i,j,k}\right]}{\sum_{i=1}^{N} \sum_{j=1}^{M} E\left[Z_{i,j,k}\right]}, \zeta_{k,0}^{(t+1)}=\frac{\sum_{i=1}^{N} \sum_{j=1}^{M} G_{i,j,k}\left(1-E\left[Z_{i,j,k}\right]\right)}{\sum_{i=1}^{N} \sum_{j=1}^{M} \left(1-E\left[Z_{i,j,k}\right]\right)}$$

Equation 14

Equation 15

Where in equation 15, the indicator variable *I*(*i*, *j*+*c*, *k*, ) is equal to 1 if X_i,j+c,k_ corresponds to the base indexed by .

Converge alternates between successive E and M steps until the motif PSSM learned converges to a stable value. The PSSM representation of the discovered motif is then returned as the output of the algorithm.

**Initial Parameters**:

The θ parameter for each genome is initialized to the average number of differences per base position between the aligned genome and the primary genome. The ζ parameters for each genome are simply initialized to 0.5. This simple initialization scheme for the gap indicator prior seems reasonable, since its final value at convergence is very insensitive to the initial guess of its value.

**Scoring Motifs Using Hypergeometric Enrichment**

Motifs produced by the Converge algorithm are scored using hypergeometric enrichment, which measures the likelihood of observing a particular number of motif-containing (positive) sequences in the input set under the null hypothesis that the sequences were selected randomly from the genome. The number of positive sequences observed in these random selections is assumed to be approximately distributed according to the hypergeometric distribution. We can then associate a *P*-value with this observation using the expression:

(*5*)

where *B* is the number of bound sequences and *G* is the total number of sequences represented on the microarray (or the genome). The quantities *b* and *g* represent the number of sequences in *B* and *G* matching the motif. The quantity -*log*_10_(p) is the hypergeometric enrichment score.

**Scanning Alignments for Motif Occurrences**

Given a motif PSSM Ψ, and a DNA sequence s, both of width w, the expected number of mismatches between a binding site emitted by the PSSM model and the sequence can be evaluated:

$$E\left[mismatches\right]=\sum_{i=1}^{w} \left(1-\Psi_{i}\left[s_{i}\right]\right)$$

Where Ψ_i_[s_i_], is the probability of observing base s_i_ at position i in the emitted binding site. For calculation of enrichment scores, the presence of a motif in a region of sequence was determined using a variation of this metric: weighted expected mismatches (WEM). The WEM score for a sequence of length w given a motif PSSM of length w is given below:

$$WEM=\frac{\sum_{k=1}^{K} \theta_{k}\sum_{i=1}^{w} I_{i,k}\left(E\left[mismatches_{i}\right]-\min\left(mismatches_{i}\right)\right)}{\sum_{k=1}^{K} \theta_{k}\sum_{i=1}^{w} I_{i,k}\left(\max\left(mismatches_{i}\right)-\min\left(mismatches_{i}\right)\right)}$$

Expected mismatches are weighted across the K aligned genomes using the θ parameters learned for the model in the EM stage of Converge, when available. In addition, at each position i the expected mismatches are weighted by the information content, I_i,k_, of the model relative to the background frequency of A, C, T, and G in each genome. The weighted mismatch score of the best matching sequence is subtracted, and the final value is normalized by the score of the worst matching sequence. The scores range between 0.0 for the best possible match, and 1.0 for the worst possible match to the model. This metric uses information about the sequences across all the aligned genomes as well as the importance of a mismatch at any given position to determine the quality of a sequence’s match to the motif model. An empirically determined cutoff of less than or equal to 0.11, found to maximize average enrichment across all motif models, was selected as the match threshold.

**Glossary of Terms**

| *N* – number of probe alignments | *M* – number of possible motif positions in an alignment |
| --- | --- |
| *P* – number of genomes | $\mathbf{\Pi}_{m}^{0}$- PWM for a motif when there is no gap in the window |
| $\mathbf{X}$ - sequence data vector indexed over N alignments, M positions, and P genomes | $\mathbf{\Pi}_{bg,k}^{1}$- 1^st^ order background distribution including gaps |
| $\mathbf{G}$ - gap indicator vector indexed over N alignments and M positions | $\mathbf{\Pi}_{bg,k}^{0}$- 1^st^ order background distribution excluding gaps |
| $\mathbf{Z}$- functional motif indicator vector indexed over N alignments, M positions, and P genomes | $\mathbf{\Pi}_{bg,k}^{(4th)}$- 4^th^ order Markov background distribution |
| $\mathbf{\Psi}$- vector of all parameters in the joint distribution | $\tilde{X}_{i,c,k}$- sequence 5-mer in alignment *i*, position *c*, genome *k*. |
| $\mathbf{Z}_{k\neq1}$- subset of $\mathbf{Z}$vector excluding the $Z_{i,j,1}$’s | $\zeta_{k,1}$- prior probability on the gap indicator in genome *k*, given that a functional motif is present |
| $\mathbf{Z}_{k=1}$- the $Z_{i,j,1}$’s | $\zeta_{k,0}$- prior probability on the gap indicator in genome *k*, given that no functional motif is present |
| $\mathbf{\Psi}^{window}$ - set of position weight matrices (PWM’s) modeling the probability of sequences in the motif window | $\theta_{k}$- prior probability on $Z_{i,j,k}$ given that a motif is present in the primary genome |
| $\mathbf{\Psi}^{background}$ - set of sequence background distributions | $\lambda$- prior on $Z_{i,j,1}$ |
| $Q_{i}=\sum_{j=1}^{M} Z_{i,j,1}$ | $\gamma=M\lambda$ |
| $\mathbf{\Pi}_{m}^{1}$ - PWM for a motif when there is a gap in the window |  |
